# Intranasal Immunization with Recombinant Hemagglutinin of Influenza A/H5 Virus Complexed with Novochizol Induces Virus-Neutralizing Antibodies and Protects Animals from Lethal Viral Challenge

**DOI:** 10.3390/pharmaceutics18060669

**Published:** 2026-05-28

**Authors:** Nadezhda B. Rudometova, Ksenia I. Ivanova, Vladislav V. Fomenko, Andrey P. Rudometov, Lyubov A. Kisakova, Denis N. Kisakov, Elena V. Yakovleva, Vladimir A. Yakovlev, Kristina P. Makarova, Danil I. Vakhitov, Mariya B. Borgoyakova, Ekaterina V. Starostina, Boris N. Zaitsev, Victoria R. Litvinova, Stepan A. Pyankov, Tatiana N. Ilyicheva, Alexander A. Ilyichev, Andrei S. Gudymo, Vasiliy Yu. Marchenko, Nariman F. Salakhutdinov, Aleksandr P. Agafonov, Larisa I. Karpenko

**Affiliations:** 1 State Research Center of Virology and Biotechnology “Vector”, Rospotrebnadzor, 630559 Koltsovo, Russia; nadenkaand100@mail.ru (N.B.R.); ivanova_ki@vector.nsc.ru (K.I.I.); orlova_la@vector.nsc.ru (L.A.K.); def_2003@mail.ru (D.N.K.); tigeeva_ev@vector.nsc.ru (E.V.Y.); yakovlev_va@vector.nsc.ru (V.A.Y.); makarova_kp@vector.nsc.ru (K.P.M.); vahitov_di@vector.nsc.ru (D.I.V.); borgoyakova_mb@vector.nsc.ru (M.B.B.); starostina_ev@vector.nsc.ru (E.V.S.); litvinova_vr@vector.nsc.ru (V.R.L.); pyankov_sa@vector.nsc.ru (S.A.P.); ilicheva_tn@vector.nsc.ru (T.N.I.); ilyichev@vector.nsc.ru (A.A.I.); gudymo_as@vector.nsc.ru (A.S.G.); marchenko_vyu@vector.nsc.ru (V.Y.M.); agafonov@vector.nsc.ru (A.P.A.); 2 N.N. Vorozhtsov Novosibirsk Institute of Organic Chemistry of the Siberian Branch of the Russian Academy of Sciences, 630090 Novosibirsk, Russia; fomenko@nioch.nsc.ru (V.V.F.); anvar@nioch.nsc.ru (N.F.S.)

**Keywords:** influenza A/H5 virus, hemagglutinin, intranasal immunization, Novochizol

## Abstract

**Background:** Avian influenza is a critical zoonotic infection threatening both the poultry industry and global public health. While traditional intramuscular vaccines elicit systemic immunity, they often fail to provide robust local protection at mucosal surfaces. There is thus significant interest in the development of mucosal avian influenza vaccines administered via the intranasal route. However, in humans, this approach is significantly hampered by the availability of safe and effective adjuvants. **Methods**: This study investigated the immunogenicity of a modified recombinant influenza A/H5 hemagglutinin (rHA/H5-modif) formulated with Novochizol, a novel chitosan-derived delivery system, administered intranasally to laboratory animals. **Results**: Our results demonstrate that mucosal immunization with the rHA/H5-modif/Novochizol complex induces potent humoral (IgG and IgA) and cell-mediated immune responses. Crucially, the formulation provided 100% survival in mice following a lethal challenge with highly pathogenic avian influenza A/H5. **Conclusions**: These findings position the rHA/H5-modif/Novochizol complex as a promising candidate for next-generation mucosal vaccines, in particular against highly pathogenic avian influenza A/H5 subtype.

## 1. Introduction

Avian influenza represents one of the most dangerous zoonotic infections, posing a significant threat to both agriculture and public health. Of particular concern are highly pathogenic avian influenza (HPAI) A/H5 viral strains, which are capable of causing large-scale epizootics among poultry and which possess zoonotic transmission potential [1,2]. In 2021, Russia reported the world’s first documented case of human infection with the A/H5N8 virus in seven poultry farm workers [3].

Vaccination remains the most effective strategy for controlling the spread of viral infections. Traditional vaccines are typically administered intramuscularly, eliciting systemic immunity but often failing to induce effective local mucosal protection. Consequently, there is considerable interest in the development of mucosal avian influenza vaccines that could be administered intranasally [4,5,6,7]. The nasal cavity serves as the primary portal of entry for infection—the initial site of contact for inhaled antigens—where mucosal immune responses are initiated, including IgA secretion. The establishment of local immunity at the respiratory mucosa is critical for the prevention of respiratory viral infections [8,9,10,11,12]. The development of mucosal vaccines capable of inducing both systemic and mucosal immunity represents a pressing challenge not only for respiratory pathogens but also for numerous enteric pathogens, sexually transmitted diseases, and oncogenic viruses that penetrate through mucosal surfaces [9].

Recombinant viral proteins are frequently employed as antigens for mucosal vaccines [13]. Protein antigens offer several substantial advantages compared to inactivated virus preparations. They are safe for individuals with allergies to chicken embryo proteins, contain minimal impurities capable of causing adverse effects, and provide economic efficiency in manufacturing with rapid production timelines. However, a significant limitation exists due to the low immunogenicity of recombinant antigens following intranasal administration. This is attributed to the unique characteristics of the mucosal membrane, which presents an aggressive and yet a tolerogenic environment [11,14]. Furthermore, continuous mucociliary clearance impedes stable drug retention and promotes rapid elimination from the organism [11].

To overcome this obstacle, specialized antigen delivery systems suitable for intranasal application are under active development. The absorption of proteins across mucosal membranes can be enhanced by formulating complexes with mucoadhesive polymers [11,15,16]. One promising and accessible natural biopolymer is chitosan—an aminopolysaccharide derived from chitin obtained from crustaceans and fungi. Chitosan exhibits pronounced mucoadhesive properties, low toxicity, and biodegradability. Several studies have demonstrated the efficacy of chitosan and its derivatives for antigen delivery, including antigens from seasonal influenza virus and A/H5N1 [17,18,19]. One promising chitosan derivative formulation is Novochizol, produced through intramolecular cross-linking of linear chitosan molecules (www.novochizol.ch).

In this study, we prepared a mucosal vaccine candidate, comprising recombinant hemagglutinin (HA) protein of influenza A/H5N8 virus as antigen, synthesized in CHO cells and purified by chromatography, and Novochizol, as a vaccine delivery system. The immunogenic properties of this vaccine candidate were investigated in laboratory animals following intranasal administration.

## 2. Materials and Methods

### 2.1. Cell Cultures and Viruses

CHO-K1 cells (Cell Culture Collection, State Research Center of Virology and Biotechnology “Vector”, Rospotrebnadzor) were used to generate the recombinant hemagglutinin rHA/H5-modif producer cell line.

The microneutralization assay was performed using MDCK-SIAT1 cells, kindly provided by the WHO Collaborating Center for Reference and Research on Influenza at the Francis Crick Institute.

For the microneutralization assay, influenza A/turkey/Stavropol/320-01/2020 (H5N8) virus (EPI1114749) was used, while the challenge infection was performed with influenza A/Astrakhan/3212/2020 (H5N8) virus (EPI1846961) (State Research Center of Virology and Biotechnology “Vector”, Rospotrebnadzor).

### 2.2. Generation of Recombinant Hemagglutinin rHA/H5-Modif Producer Cell Line

The gene encoding influenza A virus hemagglutinin (H5N8) was synthesized (Evrogen, Moscow, Russia) using a sequence based on A/turkey/Stavropol/320-01/2020 (H5N8) [20].

**Figure 1:**
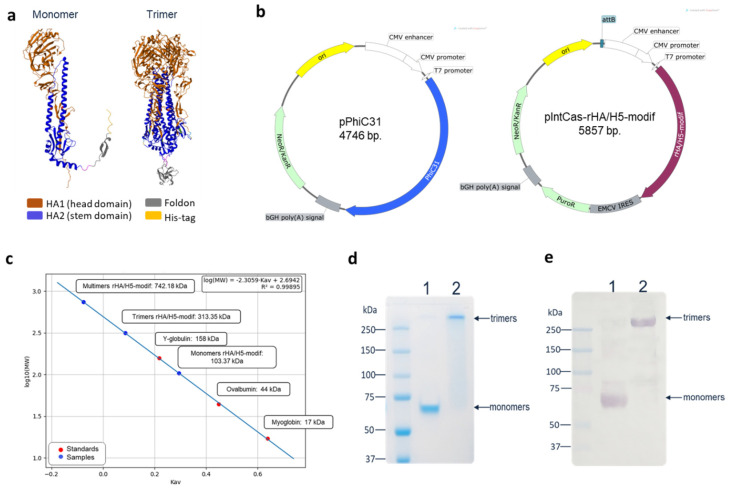
Structure and characterization of purified rHA/H5-modif protein. (**a**) the trimeric rHA/H5-modif protein model (generated with AlphaFold2); (**b**) maps of plasmid vectors used for integration, (**c**) molecular weight determination of rHA/H5-modif by gel filtration, (**d**) electropherogram of purified rHA/H5-modif protein separation in 7.5% PAGE (protein molecular weight marker Precision Plus Protein Standards (Bio-Rad, USA); 1—denaturing conditions; 2—native conditions. (**e**) Western blot analysis of purified rHA/H5-modif protein using ferret A/H5N8 immune serum: 1—denaturing conditions; 2—native conditions.

The rHA/H5-modif recombinant hemagglutinin producer cell line was generated in CHO-K1 cells using The PhiC31 Integrase System. For this purpose, two plasmid vectors were constructed: a plasmid vector carrying the PhiC31 integrase gene (pPhiC31), and a plasmid vector containing the attB site and the influenza A virus (H5N8) hemagglutinin gene in combination with EMCV-IRES-PuroR (pIntCas-rHA/H5-modif) (Figure 1b).

Subsequently, a stable rHA/H5-modif recombinant hemagglutinin producer cell line was established. One day prior to transfection, CHO-K1 cells were seeded in a 12-well plate at a density of 1 × 10^6^ cells/well in DMEM/F12 growth medium (Servicebio, Wuhan, China) supplemented with 10% fetal bovine serum (Himedia, Maharashtra, India). The following day, transfection was performed using Lipofectamine 3000 (Invitrogen, Waltham, MA, USA) with the two plasmids (pPhiC31 and pIntCas-rHA/H5-modif) at a ratio of 50:1. Several days post-transfection, the medium was replaced with fresh medium containing the antibiotic puromycin (10 μg/mL), and cells were cultured for 14 days until monolayer formation. The resulting polyclonal culture was then transferred to a T25 culture flask. After the polyclonal CHO-K1-rHA/H5-modif cell culture formed a confluent monolayer, transgene expression was analyzed by Western blot analysis. Following confirmation of transgene expression, the polyclonal culture was used for protein production.

### 2.3. Production and Purification of Recombinant Hemagglutinin rHA/H5-Modif

The CHO-K1-rHA/H5-modif producer cell line was cultured in roller bottles in DMEM/F12 medium (Servicebio, China) supplemented with 10% fetal bovine serum (Himedia, India). Upon completion of cultivation, the culture supernatant was harvested, and recombinant protein was purified by metal-affinity chromatography using IMAC Seplife FF chromatographic resin (Sunresin, Shaanxi, China). The purity of the target protein was assessed by SDS-PAGE under denaturing conditions in the presence and absence of reducing agents, followed by Coomassie G250 staining. Fractions containing the target protein were pooled and dialyzed against phosphate-buffered saline (Neofroxx, Einhausen, Germany), then concentrated using a 100 kDa cutoff centrifugal concentrator (Jet Biofil, Guangzhou, China). Subsequently, the affinity-purified protein was further purified by size-exclusion chromatography using a Chrom-LinX™ 16/1000 Tiderose GF200 column (Taidu Biotech, Suzhou, China) at a flow rate of 1 mL/min on an FPLC system. Gel Filtration Standard (Bio-Rad, Hercules, CA, USA) was used as the molecular weight standard. Molecular weight was calculated based on a standard curve generated by plotting the logarithm of molecular weight against elution volume from gel filtration.

### 2.4. Western Blot Analysis

Western blotting was carried out using the SNAP i.d. 2.0 system (Millipore, Burlington, MA, USA) following the protocol supplied by the manufacturer. As the primary antibody, ferret serum infected with influenza A/H5N8 virus was used (State Research Center of Virology and Biotechnology “Vector”, Rospotrebnadzor). As the secondary antibodies, mouse anti-ferret IgG (1:3000) (State Research Center of Virology and Biotechnology “Vector”, Rospotrebnadzor) were used. The blots were then incubated with goat anti-mouse IgG-alkaline phosphatase (1:5000) (Sigma, St. Louis, MO, USA). The immune complex was visualized by adding 1-Step™ NBT/BCIP substrate (Thermo Fisher Scientific, Waltham, MA, USA).

### 2.5. Preparation of rHA/H5-Modif-Novochizol Protein Complexes

Novochizol™ (registered international trademark Novochizol No. 1540749, US Patent Office No. 6297647) was provided by NOVOCHIZOL SA (Montey, Switzerland). Novochizol solution was prepared by sequential dissolution with ultrasonic agitation using a disperser (UZTA-0.4/22-OM, U-sonic, Biysk, Altai Krai, Russia) at maximum power in 0.15 M NaCl containing 0.5% succinic acid and 1% Novochizol. The resulting solution was sterilized by filtration through 0.45 μm apyrogenic cellulose acetate filters (Minisart^®^, Sartorius Stedim Biotech, Göttingen, Germany). Complexes of recombinant rHA/H5-modif protein with Novochizol were prepared in 0.15 M NaCl by mixing the protein solution with Novochizol at a mass ratio of 1:1. The mixture was vortexed for 10 min. The resulting particles were stored at 4 °C.

Characterization of the obtained complexes was performed using dynamic light scattering (DLS) with a Zetasizer Nano ZS Plus (Malvern Instruments, Malvern, UK). DTS1070 cuvettes were used for measurements. Surface charge was analyzed by zeta potential measurement. All measurements were performed in triplicate at 25 °C.

### 2.6. Electron Microscopy of rHA/H5-Modif/Novochizol Complex

To assess the evidence, size and shape of rHA/H5-modif/Novochizol complex, the suspension was deposited onto copper electron microscopy grids pre-coated with a carbon-stabilized film. The samples were stained using a 2% aqueous uranyl acetate. Imaging was conducted on a JEM-1400 electron microscope (Jeol, Tokyo, Japan). Digital images were captured with a Veleta digital camera (EMSIS GmbH, Münster, Germany).

### 2.7. Immunization of Laboratory Animals

Animal experiments were conducted in accordance with the legislation of the Russian Federation and the bioethical principles of the European Convention for the Protection of Vertebrate Animals Used for Experimental and Other Scientific Purposes (Strasbourg, 1986). All experiments were approved by Bioethical Protocol No. 1 (21 March 2023) issued by the Bioethics Committee of the State Research Center of Virology and Biotechnology “Vector”, Rospotrebnadzor.

Experiments were conducted on female BALB/c mice weighing 16–18 g. The animals were kept under standard laboratory conditions, with food and water available. At the first stage, the dose-dependent effect was investigated. BALB/c mice were split into four groups (*n* = 6 per group) and received two intranasal immunizations with a 3-week interval. The first group received recombinant protein complexed with Novochizol at a 1:1 ratio (2.5/2.5 μg) in a total volume of 25 μL. The second group received recombinant protein complexed with Novochizol at a 1:1 ratio (25/25 μg) in a total volume of 25 μL. The third group received 25 μg of purified recombinant hemagglutinin rHA/H5 dissolved in saline in a total volume of 25 μL. The fourth group received 25 μg of Novochizol in a total volume of 25 μL.

At the next stage, the cellular immune response and protective properties of a dose of 25 μg were investigated. BALB/c mice were split into four groups (*n* = 16 per group) and received two intranasal immunizations with a 3-week interval. The first group received 25 μg of purified recombinant hemagglutinin rHA/H5 dissolved in saline in a total volume of 25 μL. The second group received recombinant protein complexed with Novochizol at a 1:1 ratio (25/25 μg) in a total volume of 25 μL. The third group received 25 μg of Novochizol in a total volume of 25 μL. The fourth group consisted of intact (naïve) animals. For animal immobilization, inhalation anesthesia was applied (RWD Life Science, Sugar Land, TX, USA) using 2.5% isoflurane solution for 4–5 min. Subsequently, 12.5 μL of the preparation was administered into each nostril using an automatic pipette. 14 days after both the first and second immunizations, blood samples were obtained from the retro-orbital sinus. The collected blood was incubated at 37 °C for 1 h, then at 4 °C for 2 h. Following this, the samples were centrifuged at 7000× *g* for 10 min to collect the serum. The serum was then heat-inactivated by incubating it at 56 °C for 30 min and stored at −20 °C.

### 2.8. Enzyme-Linked Immunosorbent Assay (ELISA)

Enzyme-linked immunosorbent assay (ELISA) was performed according to the methodology described by Rudometova et al. [21]. Briefly, purified recombinant rHA/H5-modif protein was used as the antigen. Sera from immunized mice were titrated using a series of two-fold dilutions. The levels of IgG and IgA antibodies were measured using specific conjugates: anti-mouse IgG and anti-mouse IgA (Sigma, USA), respectively.

### 2.9. Microneutralization Assay

The microneutralization assay was carried out based on the method described by Rudometova et al. [21]. In brief, each virus stock was adjusted to contain 100 TCID_50_ per 100 μL. Two-fold serial dilutions of serum samples (200 μL) were combined with an equal volume (200 μL) of the diluted influenza virus. The mixture was then incubated for 1 h at 37 °C in a 5% CO_2_ atmosphere. Serum from unimmunized mice served as the negative control, while ferret control serum (State Research Center of Virology and Biotechnology “Vector,” Koltsovo, Russia) was used as a positive control. Next, 200 μL of the virus–serum mixture was transferred to culture plate wells containing MDCK-SIAT1 cells. After a 60 min incubation, the inoculum was removed, and the cells were washed with culture medium. The cells were then cultured for 3 days in Opti-MEM I medium supplemented with 1 μg/mL TPCK-trypsin (Sigma-Aldrich, USA) at 37 °C in 5% CO_2_. Following this, the cells were stained with a crystal violet solution, washed with water, and examined using an Agilent BioTekCytation 5 multi-mode reader (Thermo Fisher Scientific, USA) to visualize the cells. Each test was performed in triplicate. The neutralizing titer was determined as the highest serum dilution that protected 50% of the cells. In all negative control samples, cell survival was below 5%.

### 2.10. Splenocyte Isolation

Following euthanasia, spleens were collected from immunized mice and homogenized through 70 μm and 40 μm cell strainers (Jet BIOFIL, Guangzhou, China). Lysis of erythrocytes was performed with ASA buffer (Gibco, Waltham, MA, USA), followed by washing of cells in RPMI 1640 medium supplemented with 10% fetal bovine serum, 2 mM L-glutamine, and 50 μg/mL gentamicin (PanEco, Moscow, Russia). Prior to analysis, viable splenocytes were counted using a TC20™ automated cell counter (Bio-Rad, USA).

### 2.11. Intracellular Cytokine Staining (ICS) Assay

Splenocytes isolated from immunized mice were plated in a 96-well plate at a concentration of 1 × 10^6^ cells per well and stimulated for 3 h with a mixture of peptides specific for the hemagglutinin of the influenza A/H5N8 virus (A/turkey/Stavropol/320-01/2020) at a final concentration of 20 μg/mL of each peptide (TYNAELLVL, LYDKVRLQL, SFFRNVVWL, SPYQGAPSF, LYKNPTTYISVGTSTLNQ, VDTIMEKNVTVTHAQDILEK, SSWPNHETSLGVSAASPYQ, MPFHNIHPL, AGWLLGNPM, CYPGSLND, RVPEWSYI, LRNSPLREKRRKRGL, YVKSNKLVL). Peptides were selected for BALB/c mice and recognized by major histocompatibility complex class I (H-2-Dd, H-2-Kd, H-2-Ld) and class II (H2-IAd, H2-IEd) molecules. The peptides were synthesized at AtaGenix Laboratories (Wuhan, China) with a peptide purity of >95%. Brefeldin A (BioLegend, San Diego, CA, USA) was then added at 1 μg/mL, and incubation was continued for an additional 12 h at 37 °C in a humidified incubator with 5% CO_2_. A cell sample stimulated with 10 ng/mL PMA and 1 μg/mL ionomycin (BioLegend, USA) was used as a positive control to assess non-specific stimulation. After incubation, commercial monoclonal antibodies from BioLegend (USA) were used to stain surface markers: anti-CD3 (clone 500A2), anti-CD4 (clone GK1.5), and anti-CD8 (clone 53-6.7), conjugated with AF700, BV785, and FITC, respectively. To detect intracellular cytokines, monoclonal antibodies against IFN-γ (clone XMG1.2), TNF-α (clone MP6-XT22) conjugated with APC and BV650, respectively, were added to the cells. Cells were treated with a mixture of labeled monoclonal antibodies according to the manufacturer’s instructions. Samples were analyzed using a ZE5 Bio-Rad flow cytometer, and results were processed using Everest software (v3.2.12.0).

### 2.12. Viral Challenge Study

The protective efficacy study was conducted in accordance with Sanitary Rules and Regulations SanPiN 3.3686-21 of the Russian Federation [22].

At 14 days post-second immunization, mice were infected intranasally with influenza A/Astrakhan/3212/2020 (H5N8) virus at a dose of 20 LD_50_, which corresponded to 6.65 log_10_ EID_50_ (embryonic infectious dose). Challenge was performed under anesthesia using a combination of Zoletil 100 (Delpharm Tours, Chambray-lès-Tours, France) and Xyla (Interchemie, Venray, The Netherlands).

After the challenge, mice were observed daily for 14 days. Any clinical signs of disease were recorded, such as ruffled fur, decreased body temperature, weight loss, neurological symptoms, and death. Animals showing severe conditions likely to result in death, including a loss of more than 20% of initial body weight or lethargy, were euthanized by cervical dislocation. At the conclusion of the study, all remaining mice were humanely euthanized using the same method.

### 2.13. Statistical Analysis

Data analysis was carried out using GraphPad Prism 9.0 software (GraphPad Software, Inc., San Diego, CA, USA). Quantitative data were expressed as medians with ranges and assessed using non-parametric tests. Comparisons between two independent groups were made with the Mann–Whitney U test. Survival curves were constructed using the Kaplan–Meier method, and differences in survival rates between the experimental and control groups were evaluated using the Mantel–Cox (log-rank) test.

## 3. Results

### 3.1. Production, Purification and Characterization of rHA/H5-Modif

The recombinant immunogen, designated rHA/H5-modif, was designed based on the A/turkey/Stavropol/320-01/2020 (H5N8) sequence. To enhance stability and trimerization, the sequence was modified by removing transmembrane and cytoplasmic domains; introducing amino acid substitutions (H25R, H26W, H106R, K51I, E103I) in the protease-vulnerable pH-switch region; adding a T4 trimerization domain; adding a poly-His tag to facilitate subsequent purification.

Figure 1a shows a model of rHA/H5-modif obtained using the AlphaFold2 program.

The gene encoding rHA/H5-modif was codon-optimized for expression in mammalian cells and cloned into the plasmid integration vector pIntCas-rHA/H5-modif. The rHA/H5-modif recombinant hemagglutinin producer cell line was generated in CHO-K1 cells using the PhiC31 Integrase System by transfection with both pPhiC31 and pIntCas-rHA/H5-modif plasmids (Figure 1b).

Upon completion of producer cell line cultivation, the culture supernatant was harvested, and recombinant protein purification was performed by metal-affinity chromatography followed by gel filtration. The purified rHA/H5-modif protein preparation was analyzed by SDS-PAGE under reducing (with β-mercaptoethanol) and non-reducing conditions. As shown in Figure 1d, under reducing conditions, the mobility of rHA/H5-modif protein corresponded to the theoretically calculated molecular weight of the monomer (~70 kDa), while under non-reducing conditions, its mobility corresponded to the molecular weight of the trimer (~250 kDa). The molecular weight and oligomeric status of the purified rHA/H5-modif protein were confirmed by size-exclusion chromatography (Figure 1c, and Supplementary Materials). The results showed that the majority of the protein in solution existed in trimeric form.

Western blotting demonstrated that both monomers and trimers of rHA/H5-modif were recognized by serum from ferrets infected with influenza A/H5N8 virus, confirming the antigenic properties of the recombinant hemagglutinin (Figure 1e).

### 3.2. Preparation of Novochizol-Recombinant Protein Complexes

Novochizol is a chitosan derivative, a natural polysaccharide. It has a globular structure due to intramolecular cross-linking of linear chitosan molecules (Novochizol SA, Monthey, Switzerland).

**Figure 2:**
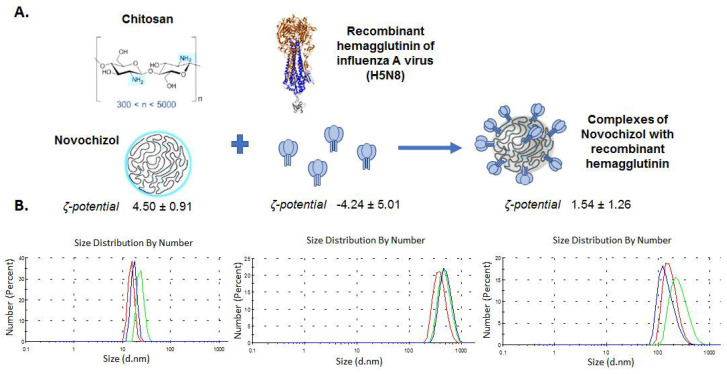
Preparation and Characterization of Recombinant Hemagglutinin rHA/H5-modif Complexes with Novochizol. (**A**) Schematic representation of rHA/H5-modif/Novochizolmicroparticle preparation. (**B**) Particle size distribution of Novochizol and rHA/H5-modif/Novochizol complexes (DLS measurements).

Complexes of recombinant protein with Novochizol were formed by mixing the recombinant protein with Novochizol in saline at a mass ratio of 1:1 (Figure 2A). The resulting complexes were characterized using dynamic light scattering (DLS), revealing a near-neutral surface charge (zeta potential consist of 1.54 mV) (Figure 2B). The particle size distributions of rHA/H5-modif/Novochizol complexes based on DLS results are shown in Figure 2B. Formation of rHA/H5-modif/Novochizol complexes was also confirmed by electron microscopy (Figure S1).

### 3.3. Immunogenicity of rHA/H5-Modif/Novochizol Complexes

**Figure 3:**
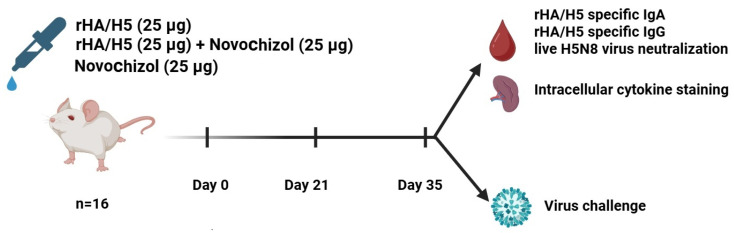
Schematic Representation of Laboratory Animal Immunization Schedule.

Immunogenic properties were investigated following intranasal immunization of BALB/c mice, as described in Section 2. Animals were divided into four groups (*n* = 16 per group) and immunized intranasally twice with a 3-week interval. A 25 μg protein dose per animal was administered each time. The immunization schedule for laboratory animals is presented in Figure 3. Additionally, a dose of 2.5 μg in the same ratio of rHA/H5-modif/Novochizol was studied. In the case of 2.5 μg, no specific antibodies were detected in ELISA. Similar data was obtained in the microneutralization assay. The data is given in Figure S2.

The immunogenicity of the obtained rHA/H5-modif/Novochizol complexes was evaluated through their ability to induce virus-specific antibodies (IgG and IgA) as well as through T-cell responses 14 days after the second immunization.

**Figure 4:**
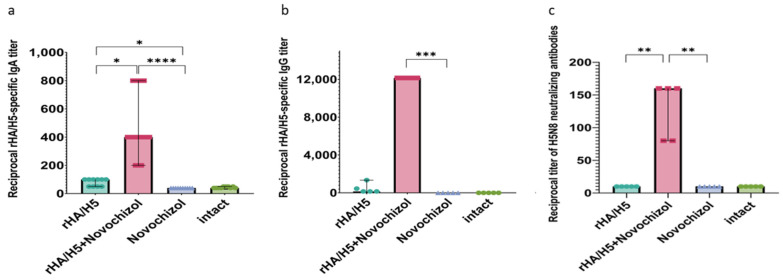
Analysis of Sera from Immunized Mice by ELISA and Microneutralization Assay. (**a**) Specific IgA antibody titers. (**b**) Specific IgG antibody titers. (**c**) Neutralizing antibody titers measured by microneutralization assay. The neutralization assay was performed on MDCK-SIAT1 cell culture using influenza A/turkey/Stavropol/320-01/2020 (H5N8) virus strain. Data are presented as median with range. Statistical analysis was performed using GraphPad Prism 9.0. software. * *p* < 0.05, ** *p* < 0.01, *** *p* < 0.001, **** *p* < 0.0001, calculated by non-parametric Mann–Whitney U-test.

The rHA/H5-modif/Novochizol complex administered intranasally was found to induce the formation of a specific humoral immune response, including both IgG and IgA antibodies, with median titers of 1:12,000 and 1:400, respectively (Figure 4a,b). Furthermore, sera were tested in a microneutralization assay using the homologous influenza A/turkey/Stavropol/320-01/2020 (H5N8) virus strain and demonstrated the ability to neutralize the virus at mean dilutions of 1:150 (Figure 4c). Antibody titers were at the level of the negative control in animals immunized with only Novochizol and with only rHA/H5-modif protein. The mice in all groups showed no signs of stress during and after the immunization procedure, and the animals’ condition was satisfactory.

### 3.4. Cellular Immune Response

**Figure 5:**
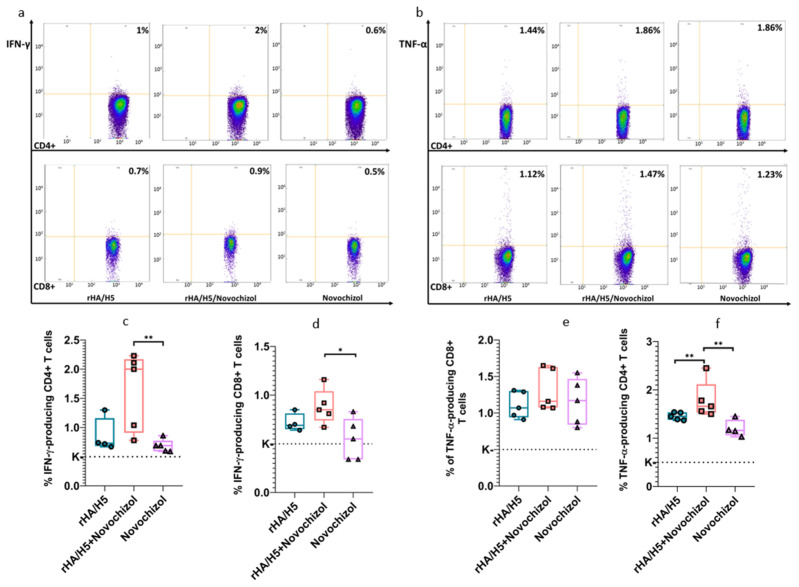
T-cell Response. (**a**,**b**) Two-parameter histogram of the distribution of T cells expressing cytokines IFN-y and TNF-α. (**c**–**f**) Evaluation of cytokine-producing CD4+ and CD8+ T-cells from spleens of BALB/c mice immunized with rHA/H5-modif/Novochizol complex using intracellular cytokine staining and flow cytometry. Group 1—mice immunized with recombinant hemagglutinin rHA/H5-modif; Group 2—mice immunized with rHA/H5-modif/Novochizol complex; Group 3—mice immunized with Novochizol in saline. Data are presented as median and range. Statistical analysis was performed using GraphPad Prism 9.0 software. * *p* < 0.05, ** *p* < 0.01, calculated by non-parametric Mann–Whitney U-test.

T-cell responses were evaluated using ICS assay. To determine cytokine-secreting T-cells in immunized mice, splenocytes were obtained. IFN-γ-and TNF-α-secreting cells were detected following stimulation of splenocytes with virus-specific peptides selected for high predicted affinity binding to MHC molecules of BALB/c mice. ICS analysis demonstrated that mice in Group 2 exhibited higher numbers of CD4+ and CD8+ IFN-γ and TNF-α-producing cells compared to Groups 1 and 3 (Figure 5c–f).

### 3.5. Protectivity Study

**Figure 6:**
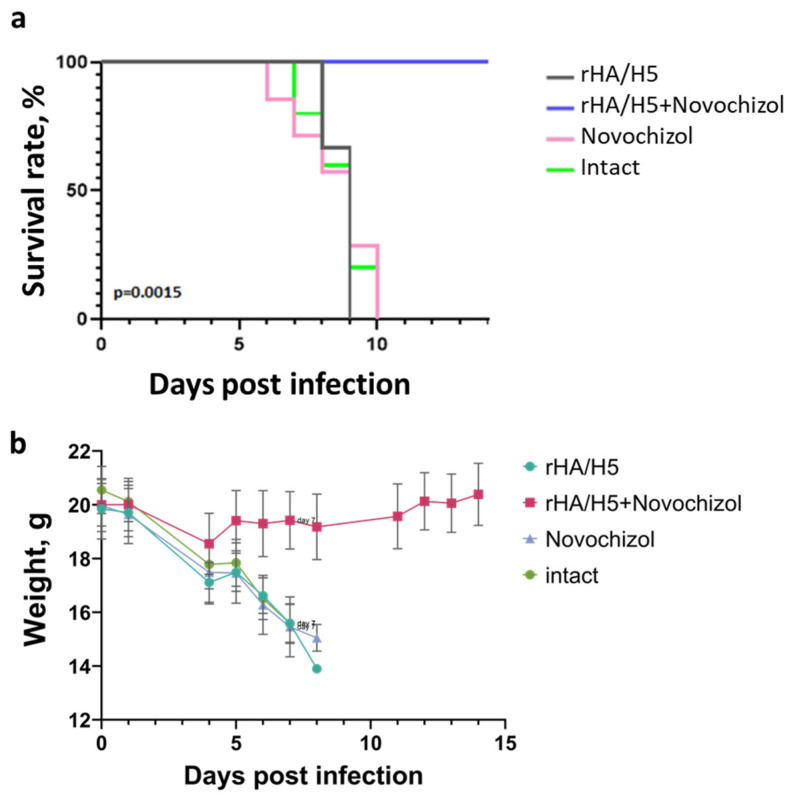
(**a**) Survival Curves of Immunized Animals Following Challenge with 20 LD_50_ of Influenza A/Astrakhan/3212/2020 (H5N8) Virus Strain. *Y*-axis: percentage of surviving animals. *X*-axis: days post-challenge. Differences in survival between study groups were statistically significant by Mantel–Cox test (*p* = 0.0015). (**b**) Graph of weight loss. *Y*-axis: animal body weight. *X*-axis: days post-challenge. Data are presented as mean with standard deviation.

The protectivity study was conducted by challenging immunized animals with live influenza A/Astrakhan/3212/2020 (H5N8) virus. A 100% survival rate was observed in the group immunized intranasally with recombinant protein complexed with Novochizol. In contrast, animals in all other groups died from the disease (Figure 6a). The weight loss graph is shown in Figure 6b.

## 4. Discussion

Avian influenza virus, like other respiratory viruses, is capable of entering the organism through the respiratory mucosa. Therefore, to prevent respiratory infectious diseases, it is important to establish local mucosal immunity, which can be achieved through mucosal immunization. Direct delivery of antigens to the respiratory mucosa can induce both systemic and local immune responses. However, insufficient immune response intensity is often observed following mucosal administration of recombinant or inactivated vaccines. Mucosal adjuvants may solve this problem by significantly enhancing antigen immunogenicity and strengthening the immune response [23]. However, the development of human mucosal vaccines remains substantially challenging due to the limited availability of safe and effective adjuvants [24].

In this study, we investigated whether Novochizol, a chitosan derivative and a promising delivery system for various molecules [25,26,27,28,29,30], may act as an effective mucosal adjuvant. Due to its globular structure and high degree of deacetylation, Novochizol has several advantages over linear chitosan: enhanced solubility in aqueous solutions, chemical stability, resistance to biodegradation, high adhesiveness, and tissue penetration capability. These properties enable Novochizol to sorb various substances and slowly release them within tissues. The safety of Novochizol was confirmed during preclinical trials.

As an antigen for mucosal vaccine development, we used recombinant hemagglutinin (HA) protein of influenza A (H5N8) virus. HA is a promising vaccine candidate antigen: it is the major surface protein of influenza virus and serves as the target for virus-neutralizing antibodies. HA is present on the surface of viral particles as a trimer composed of monomers containing a highly variable immunogenic globular head domain (HA1) and a conserved stalk domain (HA2) [31].

In this study, we produced recombinant hemagglutinin (HA) protein of influenza A (H5N8) virus. To obtain a more stable structure, the recombinant HA underwent the following modifications: i, the transmembrane and cytoplasmic domains were deleted from the native sequence; ii, amino acid substitutions were introduced in the pH-switch region vulnerable to proteases (H25R, H26W, H106R, K51I, and E103I), and iii, a T4 trimerization domain was added to the C-terminus to stabilize the trimeric structure. In our previous studies we demonstrated that such modified HA, as part of DNA and mRNA vaccines administered intramuscularly to mice via jet injection, induced neutralizing antibodies and protected against lethal viral challenge [32,33]. rHA/H5-modif was expressed in CHO cells and purified by chromatography. The protein was shown to exist predominantly in trimeric form in solution (Figure 1c). rHA/H5-modif/Novochizol protein complexes were characterized using DLS. The mean particle size was 245 ± 119.5 nm, and the zeta potential consists of 1.54 mV ±1.26. Formation of rHA/H5-modif/Novochizol complexes was also confirmed by electron microscopy (Figure S1).

Assessment of the immunogenicity of the rHA/H5-modif/Novochizol complex in mice demonstrated that intranasal administration induced significantly higher titers of virus-specific antibodies—both IgG and IgA (Figure 4a,b)—compared to administration of the recombinant protein without adjuvant. In the microneutralization test, sera from immunized animals exhibited virus-neutralizing activity even at a 1:150 dilution (Figure 4c). These data indicate that Novochizol facilitates more efficient delivery of rHA/H5-modif to mucosal immune compartments, enhancing antigen uptake, dendritic cell activation, and subsequent B-cell priming. The concomitant induction of systemic IgA alongside IgG further supports the ability of the adjuvant to stimulate both systemic and mucosal-associated humoral immunity.

Within this study, two antigen payloads of the rHA/H5-modif/Novochizol complex (2.5 μg and 25 μg) were tested at a 1:1 ratio. Using ELISA and microneutralization assays, specific antibodies were not detected at the low dose; therefore, the 25 μg dose was selected for further experiments. Despite the high immunogenicity and protective efficacy of this dose, the obtained results underscore the need for a more detailed investigation of the dose-dependent effect for both the antigen and the adjuvant. In particular, optimization of the antigen–adjuvant ratio and examination of intermediate doses will be critically important for determining the minimum effective dose and enhancing the translational scalability of this formulation.

Furthermore, we investigated T-cell immunity in response to complex administration, as accumulating evidence emphasizes the critical role of T-cell-mediated immunity in protectivity against influenza [34,35,36].

The cytokines IFN-γ and TNF-α are crucial mediators of the protective T-cell response: they promote the activation, proliferation, and polyfunctionality of CD4^+^ and CD8^+^ T cells, which is essential for the elimination of virus-infected cells and the establishment of cross-protection. Intracellular cytokine staining (ICS) results showed that the frequency of CD4^+^ and CD8^+^ T cells secreting IFN-γ and TNF-α in response to stimulation with virus-specific peptides was significantly higher in mice immunized with the rHA/H5-modif/Novochizol complex compared to control groups (Figure 5). Importantly, TNF-α levels remained within the physiological range, indicating a developed but non-hyperinflammatory response—a favorable profile for the safety of respiratory vaccines and minimizing the risk of immunopathology.

The functional significance of the described immune parameters was confirmed in a challenge study. Immunization with the rHA/H5-modif/Novochizol complex provided 100% survival of animals following lethal challenge with the avian influenza virus strain A/Astrakhan/3212/2020 (H5N8) (Figure 6). Complete protection significantly correlated with high neutralizing antibody titers and a pronounced T-cell response, collectively indicating that the adjuvant formulation elicits a coordinated humoral and cellular response capable of suppressing viral replication and preventing disease progression.

Despite these promising results, several limitations of this work should be noted.

First, the assessment of cellular immunity focused primarily on IFN-γ and TNF-α production to characterize Th1 polarization and monitor the inflammatory background. Direct quantification of IL-4 and IL-17 was not performed, which limits a comprehensive evaluation of the Th2/Th17 balance. Given the importance of cytokine equilibrium for respiratory vaccines—where an inappropriate shift toward Th2/Th17 could potentially exacerbate lung immunopathology—we explicitly acknowledge the absence of data on these markers as a limitation of the current study and emphasize the need for broader cytokine profiling in future work.

Second, despite attempts to directly measure mucosal IgA in nasal washes, technical difficulties inherent to the mouse model—specifically, microtrauma to the highly vascularized nasal mucosa during lavage, leading to transudation of serum components (IgG contamination) and high variability—precluded the inclusion of these data in the final analysis. Consequently, mucosal immunity was assessed indirectly via systemic IgA levels and the T-cell response, which correlated significantly with complete protection against lethal infection.

Third, protective efficacy was evaluated exclusively against the A/H5N8 strain, which is highly antigenically similar to the rHA/H5-modif immunogen. Although our previous studies demonstrated the cross-protective potential of HA/H5-based immunogens in DNA and mRNA vaccine formats against heterologous H5N1 strains [20,33], the current recombinant protein formulation requires validation against a broader panel of highly pathogenic avian influenza (HPAI) strains to fully assess the breadth of cross-protection.

## 5. Conclusions

In this study, we have shown that intranasal immunization with the rHA/H5-modif/Novochizol complex induced both specific humoral responses (IgG and IgA antibodies) and cellular immune responses, leading to protective immunity. The efficacy of immunization was confirmed by challenging the animals with a lethal dose of avian influenza virus subtype A/H5, and the 100% protection observed in the immunized groups. The rHA/H5-modif/Novochizol complex appears a promising candidate for the development of an effective vaccine against highly pathogenic avian influenza subtype A/H5. More generally, given its safety profile and ease of production, Novochizol may represent an interesting option for the rapid development of vaccines against emerging highly pathogenic avian influenza viruses.

## Data Availability

The original contributions presented in this study are included in the article/Supplementary Materials. Further inquiries can be directed to the corresponding authors.

## Supplementary Materials

The following supporting information can be downloaded at: https://www.mdpi.com/article/10.3390/pharmaceutics18060669/s1, Figure S1: A—Electron micrograph of Novochizol; B—Electron micrograph of rHA/H5+Novochizol complexes. Scale bar—100 nm; Figure S2: Analysis of Sera from Immunized Mice by ELISA; Table S1: Neutralizing activity of immune sera against A/turkey/Stavropol/320-01/2020 (H5N8) virus strain (reverse titer value).
