# Supplementary material for: Intranasal Immunization with Recombinant Hemagglutinin of Influenza A/H5 Virus Complexed with Novochizol Induces Virus-Neutralizing Antibodies and Protects Animals from Lethal Viral Challenge
Source: Pharmaceutics. 2026 May 28;18(6):669. doi: 10.3390/pharmaceutics18060669 (PMC13305460; doi:10.3390/pharmaceutics18060669)
Supplement: Supplementary file 1 [file pharmaceutics-18-00669-s001.zip › pharmaceutics-4280153-supplementary.pdf]

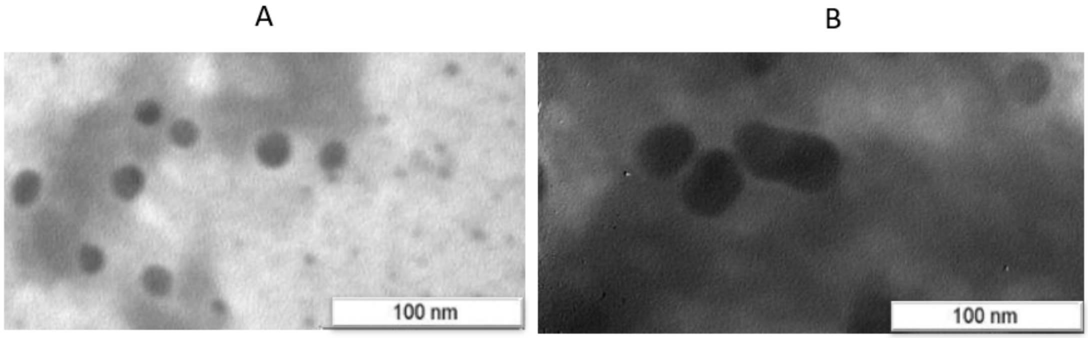

Figure S1. A – Electron micrograph of Novochizol; B – Electron micrograph of rHA/H5+Novochizol complexes. Scale bar – 100 nm.

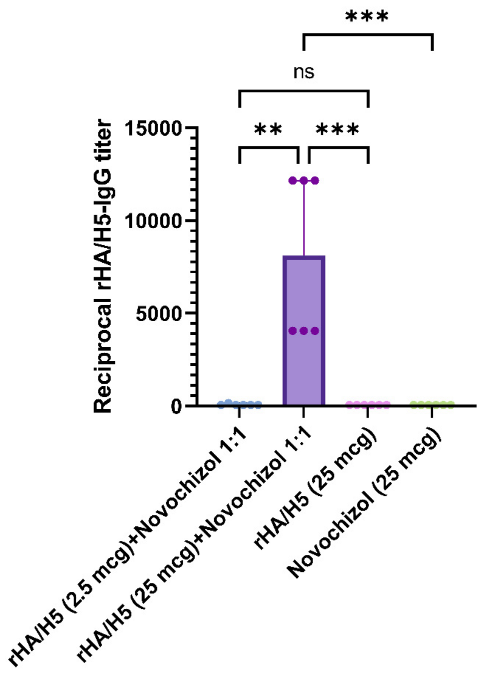

Figure S2. Analysis of Sera from Immunized Mice by ELISA. Specific IgG antibody titers. Data are presented as median with range. Statistical analysis was performed using GraphPad Prism 9.0 software. **$p < 0.01$, ***$p < 0.001$, calculated by non-parametric Mann-Whitney U-test.

Table S1. Neutralizing activity of immune sera against A/turkey/Stavropol/320-01/2020 (H5N8) virus strain (reverse titer value).

Serum №	i/n rHA/H5+Novochizol, 1:1, 2.5 µg	i/n rHA/H5+Novochizol, 1:1, 25 µg	i/n rHA/H5, 25 µg	i/n Novochizol, 25 µg
**1**	<20	320	<20	<20
**2**	<20	160	<20	<20
**3**	<20	1280	<20	<20
**4**	<20	320	<20	<20
**5**	<20	160	<20	<20
**6**	<20	320	<20	<20
